# Brown and Levy Steady-State Motions

**DOI:** 10.3390/e27060643

**Published:** 2025-06-16

**Authors:** Iddo Eliazar

**Affiliations:** School of Chemistry, Tel Aviv University, Tel Aviv 6997801, Israel; eliazar@tauex.tau.ac.il

**Keywords:** ornstein-uhlenbeck processes, levy-driven processes, markov processes and Langevin dynamics, noah effect and heavy tails, joseph effect and long-range dependence, memory and correlation, 02.50.-r (probability theory, stochastic processes, and statistics), 05.40.-a (fluctuation phenomena, random processes, noise, and Brownian motion)

## Abstract

This paper introduces and explores a novel class of Brown and Levy steady-state motions. These motions generalize, respectively, the Ornstein-Uhlenbeck process (OUP) and the Levy-driven OUP. As the OUP and the Levy-driven OUP: the motions are Markov; their dynamics are Langevin; and their steady-state distributions are, respectively, Gauss and Levy. As the Levy-driven OUP: the motions can display the Noah effect (heavy-tailed amplitudal fluctuations); and their memory structure is tunable. And, as Gaussian-stationary processes: the motions can display the Joseph effect (long-ranged temporal dependencies); and their correlation structure is tunable. The motions have two parameters: a critical exponent which determines the Noah effect and the memory structure; and a clock function which determines the Joseph effect and the correlation structure. The novel class is a compelling stochastic model due to the following combination of facts: on the one hand the motions are tractable and amenable to analysis and use; on the other hand the model is versatile and the motions display a host of both regular and anomalous features.

## 1. Introduction

The paradigmatic model—in science and engineering at large—for random motions that are in statistical steady-state is the *Ornstein-Uhlenbeck process* (OUP) [1,2,3,4]. The interest in the OUP and its applications is broad [5,6,7,8,9,10,11,12].

Almost a century after its inception, OUP research is active and ongoing, e.g.: time averages [13]; phase descriptions [14]; ergodicity breaking [15]; survival analysis [16]; first-passage area [17]; optical tweezers [18]; large deviations [19]; high-dimensionality [20]; active particles [21,22,23,24,25]; stochastic resetting [26,27,28]; and parameter estimation [29,30]. Examples of most recent OUP research include: memory effects [31]; wind profiles [32]; dynamical multimodality [33]; identification [34]; and rare events [35].

The OUP is the only random motion that is [36]: Gaussian [37,38,39] and Markov [40,41,42] *and* stationary [43,44,45]. In turn, the OUP exhibits the following amplitudal and temporal behaviors. Amplitude-wise: the OUP steady-state distribution is Gauss (Normal), and hence the OUP fluctuations are ‘mild’ [46]. Time-wise: the OUP auto-correlation is exponential, and hence the OUP dependencies are short ranged. Also, the OUP dynamics are governed by the *Langevin* stochastic differential equation [47,48,49].

The underpinning source of randomness that ‘drives’ the OUP is *Brownian motion* [50]. Shifting from Brownian motion to *Levy motion* yields the Levy-driven OUP [51,52,53,54,55,56,57,58,59,60,61,62]. As in the case of the OUP, also in the case of the Levy-driven OUP: interest and applications are broad, and the research is active and ongoing [63,64,65,66,67,68,69,70,71,72,73,74,75,76].

The shift from the OUP to the Levy-driven OUP maintains the Markov and stationary properties, and lets go of the Gaussian property. In turn, the steady-state distribution changes from Gauss (Normal) to Levy, and hence the fluctuations of the Levy-driven OUP are ‘wild’ [46]. Thus, amplitude-wise: the Levy steady-state distribution is ‘heavy tailed’ [77,78], and hence the Levy-driven OUP displays the ‘Noah effect’ [79]. Also, the Levy-driven OUP maintains the Langevin dynamics and the exponential correlation structure.

Orthogonal to the Levy approach is the Gauss approach. This approach maintains the Gaussian and stationary properties, and lets go of the Markov property. Taking on the Gauss approach, the specific exponential auto-correlation (which characterizes the OUP) is replaced by a general auto-correlation. In turn, the decay of the auto-correlation can be slow – in which case the dependencies of the resulting Gaussian-stationary motion are long ranged [80,81,82]. Thus, time-wise: the Gauss approach is capable of yielding motions that display the ‘Joseph effect’ [79].

So, on the one hand, the Levy approach affects the amplitudal behavior. And, on the other hand, the Gauss approach affects the temporal behavior. Consequently, one would expect that the former approach does not affect motion-memory, and that the latter approach does. Most recently, it was shown that—rather surprisingly and counter-intuitively—the situation is actually the other way round [76]: the Levy approach affects motion-memory, whereas the Gauss approach does not.

This paper presents a third approach for ‘tinkering’ with the OUP: a ‘golden-path’ approach that interlaces the best of the Levy and Gauss approaches. Rather than letting go of either the Gaussian property or the Markov property, the novel approach relaxes the stationary property. Specifically, stationarity is relaxed to the principle feature of the OUP: the statistical steady-state behavior. As shall be shown, the novel approach yields a versatile class of steady-state motions (SSMs) with the following features.

▶ **The SSMs can jointly display the Noah and Joseph effects.**
▶ **The SSMs’ correlation and memory structures are tunable.**
▶ **The SSMs are Markov and their dynamics are Langevin.**

On the one hand, the SSMs offer a riches of statistical behaviors which neither the Levy approach nor the Gauss approach can offer on their own. On the other hand, the SSMs are as tractable as the OUP. The novel class of SSMs is investigated here in detail. To that end, this paper shall make use of recently established results regarding Ornstein-Uhlenbeck memory [76], and regarding power Levy motion [83,84,85]. The paper is organized as follows.

Section 2 reviews the ‘pillars’ of the SSMs: Brownian motion, Levy motion, and a spatio-temporal transformation of random motions. Considering the inputs of the spatio-temporal transformation to be Brownian motion and Levy motion: Section 3 addresses the outputs’ increments; and Section 4 addresses the outputs’ correlations. Then, the paper carries on as follows: Section 5 introduces and explores the SSMs; Section 6 further investigates these motions; and Section 7 explores the motions’ Langevin dynamics. Last, Section 8 concludes with an overview. Readers looking for an ‘executive summary’ can go directly to Section 8.

## 2. Preliminaries

This section reviews three pillars on which the SSMs ‘stand’: the symmetric Levy-sable distribution (Section 2.1); the symmetric Levy-sable process (Section 2.2); and a spatio-temporal transformation of random motions (Section 2.3). As shall be described below, the symmetric Levy-sable (SLS) process is the ‘mother process’ of Brownian motion (BM) and of Levy motion (LM). The acronyms SLS, as well as BM and LM, will be frequently used in this section and along the manuscript.

### 2.1. SLS Distribution

The SLS statistical distribution [86,87,88] emerges universally via the central limit theorem and its generalizations [89]. The SLS distribution has three parameters: *m*, a real number which is the distribution’s median; *s*, a positive number which is the distribution’s scale; and an exponent λ that takes values in the range 0<λ≤2, and which is the distribution’s critical parameter.

The Fourier characterization of the SLS distribution is as follows. A real-valued random variable *R* is SLS when:

(1) $$E\left[\exp\left(i\theta \frac{R-m}{s}\right)\right]=\exp\left(-\frac{1}{\lambda }{\left|\theta \right|}^{\lambda }\right)\;,$$

where −∞<θ<∞ is the underlying Fourier variable. The Fourier transform of Equation (1) highlights the ‘center role’ of the median *m*, and the ‘scaling role’ of the scale *s*. Also, the Fourier transform of Equation (1) implies that the SLS random variable *R* is symmetric about its median.

When the SLS exponent is two, λ=2, then the SLS distribution is Gauss (Normal) with: mean *m* and variance $s^{2}$. When the SLS exponent is smaller than two, 0<λ<2, then the SLS distribution is ‘heavy-tailed’ [77,78]. Namely, the deviations of the SLS random variable *R* from its center—the median *m*—are governed by the following power-law ‘tail asymptotics’:

(2) $$\mathrm{Pr}(\left|R-m\right|>x)\underset{x\to \infty }{∼}\frac{s^{\lambda }}{x^{\lambda }}\;.$$

The tail asymptotics of Equation (2) are a quantitative manifestation of the Noah effect [79].

The only case in which the SLS distribution has a well-defined and finite variance is the Gauss case. In general, the terms $s^{\lambda }$ and *s*—the variance and the standard deviation (SD) in the Gauss case—can be addressed, respectively, as an ‘extended variance’ and an ‘extended SD’. The extended variance $s^{\lambda }$ is the enumerator of the right-hand side of Equation (2). Up to multiplicative coefficients, the extended SD *s* is a quantitative measure of the inherent randomness of the SLS distribution [46,76].

### 2.2. SLS Process

Consider a real-valued random motion with positions $L\left(\tau \right)$ (τ≥0). The motion’s increment over the time interval $\left(\tau_{1},\tau_{2}\right]$ (where $\tau_{1}<\tau_{2}$) is the displacement $L\left(\tau_{2}\right)-L\left(\tau_{1}\right)$.

The motion is a *Levy process* [90,91,92] when it initiates from the origin ($L\left(0\right)=0$ where the equality holds with probability one), and when its increments are stationary and independent. Specifically: increments that correspond to time intervals with the same temporal length ($\tau_{2}-\tau_{1}$) have the same statistical distribution; and increments that correspond to non-overlapping time intervals are independent random variables.

The motion is a SLS process when it is a Levy process, and when the statistical distribution of the position $L\left(\tau \right)$ is SLS with: median 0 and extended variance τ. When the SLS exponent is two, λ=2, then the SLS process is *Brownian motion* (BM) [50]. And when the SLS exponent is smaller than two, 0<λ<2, then the SLS process is *Levy motion* (LM). LM-based models attracted major interest in science and engineering [93,94,95,96,97,98,99,100,101,102,103,104], and are continuing to do so [105,106,107,108,109,110,111,112,113,114,115,116,117,118,119,120,121].

Due to the properties of the SLS distribution, the BM and LM increments have markedly different statistics. On the one hand, the variances of the BM increments are finite, and the distribution tails of these increments are ‘light’. On the other hand, the variances of the LM increments are either not defined or infinite, and the distribution tails of these increments are ‘heavy’. BM and LM also have markedly different trajectories: a continuous curve in the BM case; and a trajectory that evolves via jumps—and only via jumps—in the LM case.

According to the Levy-Ito decomposition theorem [90,91,92], a general Levy process is the sum of three independent parts. One part is deterministic: a linear temporal function, which manifests the process’ drift. The two other parts are stochastic: a continuous part which is BM; and a pure-jump part. The pure-jump part is LM if and only if it is a selfsimilar and symmetric process.

### 2.3. Spatio-Temporal Transformation

As in Section 2.2, consider a real-valued random motion with positions $L\left(\tau \right)$ (τ≥0). Further considering the random motion to be an ‘input’ process, transform it to an ‘output’ process by scaling both space and time. Specifically, the output is a real-valued random motion with positions

(3) $$X\left(t\right)=A\left(t\right)L\left[C\left(t\right)\right]$$

($t\geq t_{0}$). The spatio-temporal transformation of Equation (3) uses two functions: an ‘amplitude’ $A\left(t\right)$ that manifests the spatial scaling; and a ‘clock’ that manifests the temporal scaling. The amplitude is positive, and the clock is monotone increasing from $C(t_{0})\geq 0$ to $\lim_{t\to \infty }C\left(t\right)=\infty$.

Specific choices of the input, as well as of the amplitude and clock, yield specific outputs. With regard to BM and LM inputs, examples of specific outputs include: scaled BM [122,123,124,125,126,127,128,129,130,131,132,133], and scaled LM; power BM [134,135,136,137], and power LM [83,84,85]; the OUP [136], and the Levy-driven OUP [84]. The Ornstein-Uhlenbeck examples are a special case of the Lamperti transformation [138]: a general mapping of selfsimilar processes to stationary processes. A most useful stochastic tool [139,140,141,142,143,144,145,146], the Lamperti transformation itself is a special case of the spatio-temporal transformation of Equation (3).

Henceforth the input is set to be the SLS process. In turn, the output’s positions are SLS. Specifically, the position $X\left(t\right)$ is SLS with: median 0 and extended variance

(4) $$V\left(t\right)=A{\left(t\right)}^{\lambda }C\left(t\right)\;.$$

So, in the transition from the input’s positions to the output’s positions: the positions’ distributions remain SLS; their medians remain zero; and their extended variances change from τ to that of Equation (4).

## 3. Output Increments

This section addresses the statistics of the output’s increments. Specifically, the output’s increment over the time interval $\left(t_{1},t_{2}\right]$ (where $t_{0}<t_{1}<t_{2}$) is the displacement $X\left(t_{2}\right)-X\left(t_{1}\right)$.

The input is set to be the SLS process. In turn, the properties of the SLS process, and the structure of the spatio-temporal transformation of Equation (3), imply that [83]: the statistical distributions of output’s increments are SLS. Moreover, also the conditional statistical distributions of the output’s increments—given their initial positions—are SLS [84]. The medians and the extended variances of these unconditional and conditional SLS distributions are detailed in Table 1.

As evident from Table 1, the increments’ unconditional and conditional SLS distributions coincide if and only if the amplitude $A\left(t\right)$ is a flat function. When the amplitude $A\left(t\right)$ is a monotone function then there are marked differences between the increments’ SLS distributions. Indeed, shifting from the unconditional SLS distribution to the conditional SLS distribution: the median changes from zero to non-zero (whenever $X\left(t_{1}\right)\neq 0$); and the extended variance decreases.

Specifically, when the amplitude $A\left(t\right)$ is a monotone function then the extended-variance reduction (from the unconditional SLS distribution to the conditional SLS distribution) is $V\left(t_{1}\right){\left|A\left(t_{2}\right)/A\left(t_{1}\right)-1\right|}^{\lambda }$. In turn, measured relative to the extended variance of the conditional SLS distribution, the extended-variance reduction is

(5) $$Q\left(t_{1};t_{2}\right)=\frac{V\left(t_{1}\right)}{V\left(t_{2}\right)}\frac{{\left|\frac{A\left(t_{2}\right)}{A\left(t_{1}\right)}-1\right|}^{\lambda }}{1-\frac{C\left(t_{1}\right)}{C\left(t_{2}\right)}}\;.$$

The increments’ SLS distributions give rise to three informative ratios [76] which will be addressed below: a signal-to-noise ratio (Section 3.1); a noise-to-noise ratio (Section 3.2); and a variance-to-variance ratio (Section 3.3). These ratios quantify the output’s memory, and they all involve the variance-reduction quantity of Equation (5). The section ends with a short conclusion (Section 3.4).

### 3.1. Signal-to-Noise Ratio

Consider the conditional statistical distribution of the increment $X\left(t_{2}\right)-X\left(t_{1}\right)$, given the information $X\left(t_{1}\right)$. The bottom row of Table 1, together with the definition of the SLS distribution, yield the stochastic representation

(6) $${\left.X\left(t_{2}\right)-X\left(t_{1}\right)\right|}_{X\left(t_{1}\right)}\overset{Law}{=}\underset{\mathrm{Signal}\;\mathrm{coefficient}}{\underset{︸}{\left[\frac{A\left(t_{2}\right)}{A\left(t_{1}\right)}-1\right]}}\cdot \underset{\mathrm{Signal}}{\underset{︸}{X\left(t_{1}\right)}}+\underset{\mathrm{Noise}\;\mathrm{coefficient}}{\underset{︸}{{\left\{V\left(t_{2}\right)\left[1-\frac{C\left(t_{1}\right)}{C\left(t_{2}\right)}\right]\right\}}^{1/\lambda }}}\cdot \underset{\mathrm{Noise}}{\underset{︸}{R_{*}}}\;.$$

Namely, in Equation (6): the equality is in law; $R_{*}$ is a ‘standardized’ SLS random variable (i.e., with median zero m=0 and with scale one s=1); and $R_{*}$ is independent of the information $X\left(t_{1}\right)$.

The stochastic representation of Equation (6) implies that the increment $X\left(t_{2}\right)-X\left(t_{1}\right)$ is the sum of two parts—one deterministic and one stochastic. The deterministic part is the product of: (**i**) the information $X\left(t_{1}\right)$—which assumes the role of a known ‘signal’; and (**ii**) a signal coefficient. The stochastic part is the product of: (**i**) the SLS random variable $R_{*}$—which assumes the role of an unknown ‘noise’; and (**ii**) a noise coefficient.

Combined together, the signal coefficient and the noise coefficient yield a signal-to-noise ratio (SNR) $SNR\left(t_{1};t_{2}\right)$. Specifically: the SNR’s enumerator is the absolute value of the signal coefficient of Equation (6); and the SNR’s denominator is the noise coefficient of Equation (6). It follows from Equation (6) that

(7) $$SNR\left(t_{1};t_{2}\right)={\left[\frac{Q\left(t_{1};t_{2}\right)}{V\left(t_{1}\right)}\right]}^{1/\lambda }\;,$$

where $Q\left(t_{1};t_{2}\right)$ is the variance-reduction quantity of Equation (5).

### 3.2. Noise-to-Noise Ratio

Consider the unconditional statistical distribution of the increment $X\left(t_{2}\right)-X\left(t_{1}\right)$. The middle row of Table 1, together with the definition of the SLS distribution, yield the stochastic representation

(8) $$X\left(t_{2}\right)-X\left(t_{1}\right)\overset{Law}{=}\underset{\mathrm{Noise}\;\mathrm{coefficient}}{\underset{︸}{{\left\{V\left(t_{1}\right){\left|\frac{A\left(t_{2}\right)}{A\left(t_{1}\right)}-1\right|}^{\lambda }+V\left(t_{2}\right)\left[1-\frac{C\left(t_{1}\right)}{C\left(t_{2}\right)}\right]\;\right\}}^{1/\lambda }}}\cdot \underset{\mathrm{Noise}}{\underset{︸}{R_{*}}}\;.$$

Namely, in Equation (8): the equality is in law; and $R_{*}$ is a ‘standardized’ SLS random variable (i.e., with median zero m=0 and with scale one s=1).

As described in Section 3.1, the stochastic representation of Equation (6) is the sum of a deterministic part and a stochastic part. In contrast, the stochastic representation of Equation (8) comprises only of a stochastic part. Analogously to the stochastic part of Equation (6), the stochastic part of Equation (8) is the product of: (**i**) the SLS random variable $R_{*}$—which assumes the role of an unknown ‘noise’; and (**ii**) a noise coefficient.

As noted in the opening of this section (provided that the amplitude $A\left(t\right)$ is not a flat function): the shift from the unconditional distribution to the conditional distribution results in a decrease of the extended variance. Indeed, once the information $X\left(t_{1}\right)$ is given—the increment’s distribution becomes less noisy. The noise reduction is quantified by a noise-to-noise ratio (NNR) $NNR\left(t_{1};t_{2}\right)$.

Specifically, the NNR’s enumerator is the noise coefficient of Equation (6)—the scale of the increment’s conditional SLS distribution. And, the NNR’s denominator is the noise coefficient of Equation (8)—the scale of the increment’s unconditional SLS distribution. It follows from Equations (6) and (8) that

(9) $$NNR\left(t_{1};t_{2}\right)={\left[\frac{1}{1+Q\left(t_{1};t_{2}\right)}\right]}^{1/\lambda }\;,$$

where $Q\left(t_{1};t_{2}\right)$ is the variance-reduction quantity of Equation (5).

### 3.3. Variance-to-Variance Ratio

Consider the conditional distribution of the increment $X\left(t_{2}\right)-X\left(t_{1}\right)$, given the information $X\left(t_{1}\right)$. As described in Section 3.1, this conditional distribution admits the stochastic representation of Equation (6). Denote the right-hand side of Equation (6) $R_{con}$. The random variable $R_{con}$ is SLS with: median $m_{con}$ that is the deterministic part (i.e., the ‘signal’ part) of the right-hand side of Equation (6); and scale $s_{con}$ that is the noise coefficient of the right-hand side of Equation (6).

Consider the unconditional distribution of the increment $X\left(t_{2}\right)-X\left(t_{1}\right)$. As described in Section 3.2, this unconditional distribution admits the stochastic representation of Equation (8). Denote the right-hand side of Equation (8) $R_{unc}$. The random variable $R_{unc}$ is SLS with: median zero $m_{unc}=0$; and scale $s_{unc}$ that is the noise coefficient of the right-hand side of Equation (8).

Now, consider the deviations of the random variables $R_{con}$ and $R_{unc}$ from their medians. The ‘tail asymptotics’ of these deviations—in the LM-input case—are described by Equation (2): for the random variable $R_{con}$ with the median $m_{con}$ and the scale $s_{con}$; and for the random variable $R_{unc}$ with the median $m_{unc}$ and the scale $s_{unc}$. Equation (2) implies that the ratio of the tail asymptotics is

(10) $$\frac{\mathrm{Pr}\left(\left|R_{con}-m_{con}\right|>x\right)}{\mathrm{Pr}\left(\left|R_{unc}-m_{unc}\right|>x\right)}\underset{x\to \infty }{⟶}\frac{s_{con}^{\lambda }}{s_{unc}^{\lambda }}={\left(\frac{s_{con}}{s_{unc}}\right)}^{\lambda }\;.$$

The left-hand side of Equation (10) is a tail-to-tail ratio (TTR). The TTR’s enumerator is the probability tail of the increment’s conditional SLS distribution. The TTR’s denominator is the probability tail of the increment’s unconditional SLS distribution.

The middle part of Equation (10) is a ‘variance-to-variance’ ratio (VVR) $VVR\left(t_{1};t_{2}\right)$. The VVR’s enumerator $s_{con}^{\lambda }$ is the extended variance of the increment’s conditional SLS distribution. The VVR’s denominator $s_{unc}^{\lambda }$ is the extended variance of the increment’s unconditional SLS distribution.

The right-hand side of Equation (10) is the $\lambda^{\mathrm{th}}$ power of the ratio $s_{con}/s_{unc}$—which is the NNR (described in Section 3.2). So, Equations (9) and (10) imply that

(11) $$VVR\left(t_{1};t_{2}\right)=\frac{1}{1+Q\left(t_{1};t_{2}\right)}\;,$$

where $Q\left(t_{1};t_{2}\right)$ is the variance-reduction quantity of Equation (5).

As the SNR and the NNR, also the VVR is defined for the entire range of the SLS exponent (0<λ≤2). For the LM-input case (0<λ<2) this subsection presented a ‘tail perspective’ of the VVR and of the NNR – doing so via, respectively, the middle part and the right-hand side of Equation (10).

### 3.4. Conclusion

Based on the unconditional and conditional statistical distributions specified in Table 1, this section addressed three informative ratios.

▶ The signal-to-noise ratio $SNR\left(t_{1};t_{2}\right)$ of Equation (7).
▶ The noise-to-noise ratio $NNR\left(t_{1};t_{2}\right)$ of Equation (9).
▶ The variance-to-variance ratio $VVR\left(t_{1};t_{2}\right)$ of Equation (11).

The ratios quantify the output’s memory. Indeed, the ratios quantify how the knowledge of the ‘present position’ $X\left(t_{1}\right)$ affects the statistical distribution of the ‘future increment’ $X\left(t_{2}\right)-X\left(t_{1}\right)$ (where $t_{0}<t_{1}<t_{2}$). It follows from the aforementioned ratio formulae that the ratios are coupled by the relation

(12) $$VVR\left(t_{1};t_{2}\right)=NNR{\left(t_{1};t_{2}\right)}^{\lambda }=\frac{1}{1+V\left(t_{1}\right)\cdot SNR{\left(t_{1};t_{2}\right)}^{\lambda }}\;.$$

Namely, the VVR and the NNR are coupled by a power-law relation. And, the coupling of the VVR/NNR and the SNR involves the extended variance of the output’s position at the time point $t_{1}$.

## 4. Correlations

As noted in Section 2.2, the SLS process comprises two motions—Brown (λ=2) and Levy (0<λ<2)—which have markedly different behaviors. On the one hand, BM is a continuous process whose positions’ variances are finite. On the other hand, LM is a pure-jump process whose positions’ variances are either not defined or infinite.

The input is set to be the SLS process. In turn, the input behaviors are induced to the output. Consequently, in the BM-input case: the output has a well-defined covariance function—from which the output’s correlations follow. Antithetically, in the LM-input case: the output does not have a well-defined covariance function, and hence its correlations are also not well-defined (in the common statistical sense).

Being a pure-jump process, LM has an underlying jump structure. The jump structure is specified by the Levy-Ito decomposition theorem [90,91,92], and it is Poissonian [54,147]. The jump structure gives rise to a Poissonian-correlations method [148,149] that is applicable in the context of Levy-driven motions at large [150,151,152,153]. In particular—in the LM-input case–the Poissonian-correlations method is applicable to the output, and it unveils the output’s intrinsic correlation structure [85].

This section is organized as follows. Firstly, the BM-input case is addressed (Section 4.1). Thereafter, with regard to the LM-input case: the underlying jump structure is described (Section 4.2); and the Poissonian correlations are presented (Section 4.3). The section ends with a short conclusion (Section 4.4).

### 4.1. BM Input

Consider the SLS input to be BM (λ=2). In this case the input’s covariance function is $\mathrm{Cov}\left[L\left(\tau_{1}\right),L\left(\tau_{2}\right)\right]=\tau_{1}$ (where $\tau_{1}\leq \tau_{2}$) [50]. In turn, Equation (3) and the fact that the clock $C\left(t\right)$ is monotone increasing imply that: the output’s covariance function is $\mathrm{Cov}\left[X\left(t_{1}\right),X\left(t_{2}\right)\right]=A\left(t_{1}\right)A\left(t_{2}\right)C\left(t_{1}\right)$ (where $t_{1}\leq t_{2}$). With this covariance function at hand, the output’s correlations are deduced.

Denote by $F_{BM}\left(t_{1},t_{2}\right)$ the correlation of the output’s positions $X\left(t_{1}\right)$ and $X\left(t_{2}\right)$ (where $t_{1}\leq t_{2}$). Then, it follows straightforwardly from the output’s covariance function that

(13) $$F_{BM}\left(t_{1},t_{2}\right)=\sqrt{\frac{C\left(t_{1}\right)}{C\left(t_{2}\right)}}\;.$$

Section 3 addressed the conditional statistical distribution of the increment $X\left(t_{2}\right)-X\left(t_{1}\right)$ (where $t_{0}<t_{1}<t_{2}$), given the information $X\left(t_{1}\right)$. The corresponding correlation is that of the output’s position $X\left(t_{1}\right)$ and the output’s increment $X\left(t_{2}\right)-X\left(t_{1}\right)$. Denoting this correlation $G_{BM}\left(t_{1},t_{2}\right)$, a calculation that uses the output’s covariance function implies that [85]:

(14) $$G_{BM}\left(t_{1},t_{2}\right)=\frac{\frac{A\left(t_{2}\right)}{A\left(t_{1}\right)}-1}{\sqrt{1-2\frac{A\left(t_{2}\right)}{A\left(t_{1}\right)}+\frac{V\left(t_{2}\right)}{V\left(t_{1}\right)}}}\;,$$

Evidently, there is a marked difference between the two correlations. On the one hand, the correlation $F_{BM}\left(t_{1},t_{2}\right)$ is determined by the clock $C\left(t\right)$ alone, and is not affected by the amplitude $A\left(t\right)$. On the one hand, the correlation $G_{BM}\left(t_{1},t_{2}\right)$ is determined by both the amplitude $A\left(t\right)$ and the clock $C\left(t\right)$, and the amplitude’s effect on this correlation is dramatic: it determines if $G_{BM}\left(t_{1},t_{2}\right)$ is positive, negative, or zero.

### 4.2. LM Input

Consider the SLS input to be LM (0<λ<2). As noted in Section 2.2, LM is a pure-jump process. The jump structure of LM is described by the Levy-Ito decomposition theorem [90,91,92]. Specifically, the LM jumps form a Poisson point process [54,147]. In particular, the LM jumps whose sizes are greater than the positive level *l* form a ‘standard’ Poisson process with rate $c_{\lambda }/l^{\lambda }$ (where $c_{\lambda }$ is a constant that depends on the SLS exponent λ) [85].

The LM position $L\left(\tau \right)$ is the sum of all the LM jumps that occurred up to the time point τ. Following the scaling of space and time—according to the spatio-temporal transformation of Equation (3)—the input’s jump structure is induced to the output. Specifically, the output’s position $X\left(t\right)$ is as follows: it is the sum of all the LM jumps that occurred up to the time point $C\left(t\right)$, and the sizes of these jumps are multiplied by $A\left(t\right)$.

Set the focus on the summands comprising $X\left(t\right)$ whose sizes are greater than the positive level *l*, and denote their number $N_{l}\left(t\right)$. Namely, the number $N_{l}\left(t\right)$ counts the LM jumps that: (**i**) occurred up to the time point $C\left(t\right)$; and (**ii**) are greater than $l/A\left(t\right)$. In turn, the aforementioned Poisson-process fact [85] implies that the number $N_{l}\left(t\right)$ is a Poisson random variable whose mean and variance are:

(15) $$E\left[N_{l}\left(t\right)\right]=\mathrm{Var}\left[N_{l}\left(t\right)\right]=\frac{c_{\lambda }}{l^{\lambda }}\cdot V\left(t\right)\;,$$

where $V\left(t\right)$ is the extended variance of Equation (4).

The right-hand side of Equation (15) is the product of two terms: (**i**) the level-dependent term $c_{\lambda }/l^{\lambda }$, which is the aforementioned Poisson-process rate; and (**ii**) the time-dependent term $V\left(t\right)$, which is the extended variance of the output’s positions. So, Equation (15) yields a genuine variance-meaning to the extended variance of Equation (4). [Indeed, prior to Equation (15), $V\left(t\right)$ was merely an extension of the variance—rather than a genuine variance.]

Now, consider two time points $t_{1}<t_{2}$, as well as a jump that is counted by the number $N_{l}\left(t_{1}\right)$. Exploiting the Poissonian statistics of the LM input yields the following conclusion. The probability that the jump—which was counted by the number $N_{l}\left(t_{1}\right)$—will also be counted by the number $N_{l}\left(t_{2}\right)$ is [85]:

(16) $$P\left(t_{1};t_{2}\right)=\min\left\{1,{\left[\frac{A\left(t_{2}\right)}{A\left(t_{1}\right)}\right]}^{\lambda }\right\}\;.$$

The quantity of Equation (16) manifests an intrinsic ‘survival probability’ of the output.

The parameter *l* of the number $N_{l}\left(t\right)$ is, in effect, a resolution level. Note that while the mean and the variance of the number $N_{l}\left(t_{1}\right)$ depend on the resolution level, the survival probability of Equation (16) does not. So, the survival probability is ‘level free’.

### 4.3. Poissonian Correlations

Section 4.1 addressed—in the BM-input case (λ=2)—the following correlations. The correlation $F_{BM}\left(t_{1},t_{2}\right)$ of the output’s positions $X\left(t_{1}\right)$ and $X\left(t_{2}\right)$. And the correlation $G_{BM}\left(t_{1},t_{2}\right)$ of the output’s position $X\left(t_{1}\right)$ and the output’s increment $X\left(t_{2}\right)-X\left(t_{1}\right)$. Shifting from a BM input to a LM input has the following effect: the variances of the output’s positions shift from finite to either not defined or infinite. In turn, the correlations $F_{BM}\left(t_{1},t_{2}\right)$ and $G_{BM}\left(t_{1},t_{2}\right)$ cannot be carried on to the LM-input case (0<λ<2).

As described in Section 4.2, the LM input has an underlying Poissonian jump structure. This structure is induced to the output, and it gives rise to the numbers $N_{l}\left(t\right)$ ($t\geq t_{0}$)—which are Poisson random variables (with means and variances that are specified in Equation (15)). With regard to these numbers, analogues of the correlations $F_{BM}\left(t_{1},t_{2}\right)$ and $G_{BM}\left(t_{1},t_{2}\right)$ are well-defined indeed, and shall now be presented.

Denote by $F_{LM}\left(t_{1},t_{2}\right)$ the correlation of the numbers $N_{l}\left(t_{1}\right)$ and $N_{l}\left(t_{2}\right)$ (where $t_{1}\leq t_{2}$). It was established in [85] that if the amplitude $A\left(t\right)$ is monotone decreasing then

(17) $$F_{LM}\left(t_{1};t_{2}\right)=\frac{C\left(t_{1}\right)}{C\left(t_{2}\right)}\sqrt{\frac{V\left(t_{2}\right)}{V\left(t_{1}\right)}}\;.$$

Denote by $G_{LM}\left(t_{1},t_{2}\right)$ the correlation of the number $N_{l}\left(t_{1}\right)$ and of the numbers’ increment $N_{l}\left(t_{2}\right)-N_{l}\left(t_{1}\right)$ (where $t_{1}<t_{2}$). It was further established in [85] that if the amplitude $A\left(t\right)$ is monotone decreasing then

(18) $$G_{LM}\left(t_{1};t_{2}\right)=\frac{\frac{C\left(t_{1}\right)}{C\left(t_{2}\right)}\frac{V\left(t_{2}\right)}{V\left(t_{1}\right)}-1}{\sqrt{1-2\frac{C\left(t_{1}\right)}{C\left(t_{2}\right)}\frac{V\left(t_{2}\right)}{V\left(t_{1}\right)}+\frac{V\left(t_{2}\right)}{V\left(t_{1}\right)}}}\;.$$

At the close of Section 4.2 it was noted that the survival probability of Equation (16) is ‘level free’. The Poissonian correlations $F_{LM}\left(t_{1};t_{2}\right)$ and $G_{LM}\left(t_{1};t_{2}\right)$ are also ‘level free’: they do not depend on the resolution level *l*—the parameter *l* of the number $N_{l}\left(t\right)$.

### 4.4. Conclusions

This section addressed the output’s correlations. As elucidated above, there are marked differences between the two motions that comprise the SLS input: BM (λ=2) and LM (0<λ<2).

When the input is BM then the variances of its positions are finite—and hence so are the variances of the output’s positions. In turn, the following correlations of the output were derived.

▶ The positions’ correlation $F_{BM}\left(t_{1},t_{2}\right)$ of Equation (13), and the position-increment correlation $G_{BM}\left(t_{1},t_{2}\right)$ of Equation (14).

When the input is LM then the variances of its positions are either not defined or infinite—and hence so are the variances of the output’s positions. In turn, the correlations of the BM-input case cannot be ‘carried on’ to the LM-input case.

The LM has an underlying jump structure—which is induced to the output in the LM-input case. This jump structure gives rise to jump counts: numbers that count the output’s underlying jumps. In turn, the following jump-structure quantities were derived.

▶ The survival probability $P\left(t_{1};t_{2}\right)$ of Equation (16).
▶ The numbers’ correlation $F_{LM}\left(t_{1},t_{2}\right)$ of Equation (17), and the number-increment correlation $G_{LM}\left(t_{1},t_{2}\right)$ of Equation (18).

The survival probability $P\left(t_{1};t_{2}\right)$ has no parallel in the BM-input case. In the transition from the BM-input case to the LM-input case: the numbers-based correlations $F_{LM}\left(t_{1},t_{2}\right)$ and $G_{LM}\left(t_{1},t_{2}\right)$ are the ‘Levy counterparts’ of the positions-based correlations $F_{BM}\left(t_{1},t_{2}\right)$ and $G_{BM}\left(t_{1},t_{2}\right)$.

## 5. Steady-State Motions

With the general results of Section 3 and Section 4 at hand, the stage is now set to introduce and analyze this paper’s main object: the steady-state outputs of the spatio-temporal transformation of Equation (3). These outputs are henceforth termed *steady-state motions* (SSMs).

This section begins with the characterization of the SSMs (Section 5.1). Then, a quantitative analysis of the SSMs is presented (Section 5.2). The section concludes with two special cases of SSMs (Section 5.3): Ornstein-Uhlenbeck processes (OUPs); and extensions of power motions.

### 5.1. Steady State

Consider a real-valued random process whose timeline is the real line (−∞<t<∞). The process is stationary [43,44,45] when it is invariant with respect to temporal translations: t↦t+s, where *s* is a real shift parameter. In turn, when the process is stationary then it is in steady state, i.e.,: the positions of the process—at all time points—are governed by a common statistical distribution. [Steady state is often described by the condition $\frac{\partial }{\partial t}p\left(t,x\right)=0$, where: p(t,x) is the probability density function of the process’ position at the time point *t*. Informally, p(t,x) is the probability that: at the time point *t* the value of process is *x*.]

The input is set to be the SLS process. In turn, as described in Section 2.3, the statistical distribution of the output’s position $X\left(t\right)$ is SLS with: median zero; and scale $V{\left(t\right)}^{1/\lambda }$ (where $V\left(t\right)$ is the extended variance of Equation (4)). Thus, the output is in steady state if and only if the extended variance is a flat function: $V\left(t\right)=v$, where *v* is a positive ‘variance parameter’.

As noted at the opening of this section, the steady-state outputs are termed steady-state motions (SSMs). The four following statements (in which $t_{1},t_{2}\geq t_{0}$) are equivalent characterizations of SSMs.

1. Extended-variance characterization: $V\left(t_{1}\right)=V\left(t_{2}\right)$.
2. Amplitude-clock characterization: ${\left[A\left(t_{2}\right)/A\left(t_{1}\right)\right]}^{\lambda }=C\left(t_{1}\right)/C\left(t_{2}\right)$.
3. Ratios characterization: $VVR\left(t_{1};t_{2}\right)=NNR{\left(t_{1};t_{2}\right)}^{\lambda }=1/\left[1+vSNR{\left(t_{1};t_{2}\right)}^{\lambda }\right]$.
4. Levy characterization: $P\left(t_{1};t_{2}\right)=F_{LM}\left(t_{1};t_{2}\right)=C\left(t_{1}\right)/C\left(t_{2}\right)$.

The equivalence of the characterizations follows straightforwardly from: Equation (4); Equation (12); and Equations (16) and (17). The characterizations #1 to #3 apply to the entire range of the SLS exponent (0<λ≤2). The Levy characterization applies only to the LM-input case (0<λ<2).

The following conclusions hold with regard to SSMs.

1. As the clock $C\left(t\right)$ is monotone increasing: the amplitude $A\left(t\right)$ is monotone decreasing.
2. The SNR and NNR/VVR are inversely related: the larger the SNR—the smaller the NNR/VVR; the smaller the SNR—the larger the NNR/VVR.
3. In the BM-input case, the correlations of Section 4.1 are coupled by the relation $G_{BM}\left(t_{1};t_{2}\right)=-\sqrt{[1-F_{BM}\left(t_{1},t_{2}\right)]/2}$.
4. In the LM-input case, the correlations of Section 4.3 are coupled by the relation $G_{LM}\left(t_{1};t_{2}\right)=-\sqrt{[1-F_{LM}\left(t_{1},t_{2}\right)]/2}$.

Conclusion #1 follows straightforwardly from Equation (4). Conclusion #2 follows straightforwardly from the ratios characterization. The derivations of conclusions #3 and #4 are detailed in the Appendix A.

### 5.2. Quantitative Analysis

General outputs of the spatio-temporal transformation of Equation (3) were analyzed via eight informative quantities. These quantities were presented in Section 3 and Section 4, and are the following.

With regard to the BM-input case: (**1**) the positions’ correlation $F_{BM}\left(t_{1},t_{2}\right)$ of Equation (13); and (**2**) the position-increment correlation $G_{BM}\left(t_{1};t_{2}\right)$ of Equation (14). With regard to the LM-input case: (**3**) the survival probability $P\left(t_{1};t_{2}\right)$ of Equation (16); (**4**) the numbers’ correlation $F_{LM}\left(t_{1};t_{2}\right)$ of Equation (17); and (**5**) the number-increment correlation $G_{LM}\left(t_{1};t_{2}\right)$ of Equation (18). With regard to both the BM-input and the LM-input cases, and based on the ‘mother quantity’ $Q\left(t_{1},t_{2}\right)$ of Equation (5): (**6**) the signal-to-noise ratio $SNR\left(t_{1};t_{2}\right)$ of Equation (7); (**7**) the noise-to-noise ratio $NNR\left(t_{1};t_{2}\right)$ of Equation (9); and (**8**) the variance-to-variance ratio $VVR\left(t_{1};t_{2}\right)$ of Equation (11).

With no loss of generality, consider the positions of the SSMs to be standardized, i.e., with median zero m=0 and with scale one s=1. So, the amplitude is $A\left(t\right)=C{\left(t\right)}^{-1/\lambda }$, and hence the SSMs have two parameters: the exponent λ of SLS input, which is a one-dimensional parameter; and the clock $C\left(t\right)$ of the spatio-temporal transformation, which is an infinite-dimensional parameter.

Substituting the amplitude $A\left(t\right)=C{\left(t\right)}^{-1/\lambda }$ into the aforementioned formulae yields the following conclusion: all eight quantities depend on the clock $C\left(t\right)$, and they do so via the clock ratio $r=C\left(t_{1}\right)/C\left(t_{2}\right)$. As the clock C(t) is positive and monotone increasing over the temporal range $t>t_{0}$, note that: when $t_{0}<t_{1}<t_{2}$ then the clock ratio takes values in the unit-interval range 0<r<1.

**Figure 1:**
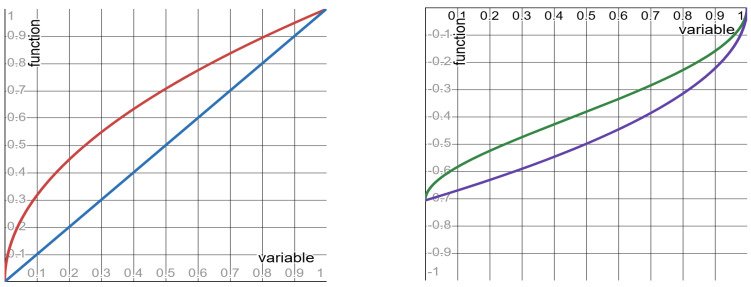
The functions ϕ(r) specified in rows #1 to #5 of Table 2. **Left panel**: red curve—row #1; blue curve—rows #3 and #4. **Right panel**: green curve—row #2; purple curve—row #5.

Quantities #1 to #5 depend on the clock $C\left(t\right)$ alone (they do not depend on the SLS exponent λ). So, these quantities admit the functional form $\phi \left(r\right)$. The shape of the functions $\phi \left(r\right)$ is monotone increasing. Per each of these quantities, Table 2 specifies: the function $\phi \left(r\right)$ and its limit values (as the clock-ratio *r* approaches the endpoints of its unit-interval range). Graphs of the functions $\phi \left(r\right)$ are depicted in Figure 1.

**Figure 2:**
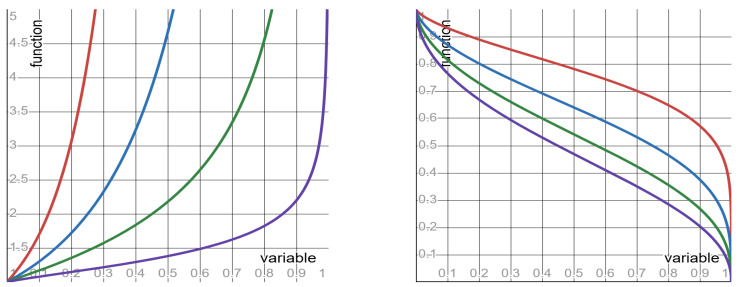
The function $\phi_{\lambda }\left(r\right)$ specified in row #6 of Table 3. **Left panel**: red curve λ = 0.2; blue curve λ = 0.4; green curve λ = 0.6; purple curve λ = 0.8. **Right panel**: red curve λ = 1.2; blue curve λ = 1.4; green curve λ = 1.6; purple curve λ = 1.8.

**Figure 3:**
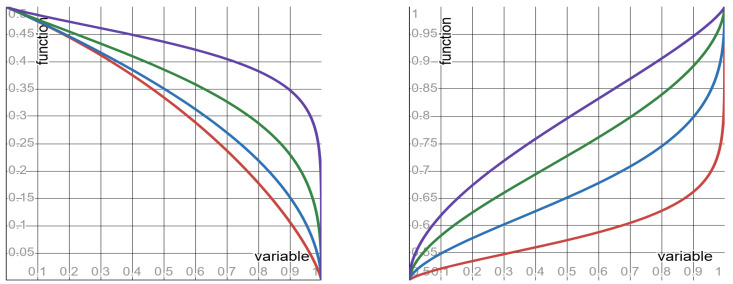
The function $\phi_{\lambda }\left(r\right)$ specified in row #8 of Table 3. **Left panel**: red curve λ = 0.2; blue curve λ = 0.4; green curve λ = 0.6; purple curve λ = 0.8. **Right panel**: red curve λ = 1.2; blue curve λ = 1.4; green curve λ = 1.6; purple curve λ = 1.8.

Quantities #6 to #8, as well as their mother quantity, depend on both the clock $C\left(t\right)$ and the SLS exponent λ. So, these quantities admit the functional form $\phi_{\lambda }\left(r\right)$ (in which the SLS exponent λ assumes the role of the form’s parameter). Depending on the value of the parameter λ, the functions $\phi_{\lambda }\left(r\right)$ display three different shapes: monotone increasing; monotone decreasing; and flat. Per each of these quantities, Table 3 specifies: the function $\phi_{\lambda }\left(r\right)$, its shape, and its limit values (as the clock-ratio *r* approaches the endpoints of its unit-interval range). Graphs of the functions $\phi_{\lambda }\left(r\right)$ of quantities #6 and #8 are depicted, respectively, in Figure 2 and Figure 3.

### 5.3. Special Cases

As described in Section 5.2, the clock ratio $r=C\left(t_{1}\right)/C\left(t_{2}\right)$ assumes a key role in the context of quantifying various statistics of the SSMs. Two special cases of the clock ratio shall now be highlighted. These special cases correspond, respectively, to two special SSMs.

**Exponential clock and OUPs**. The clock ratio $C\left(t_{1}\right)/C\left(t_{2}\right)$ is a function of the temporal difference $t_{2}-t_{1}$ alone—rather than a function of the time points $t_{1}$ and $t_{2}$—if and only if the clock is an exponential function: $C\left(t\right)=\exp\left(\gamma t\right)$, where γ is a positive exponent (as the clock is monotone increasing). In turn, the exponential clock produces the OUPs: the ‘regular’ OUP in the BM-input case; and the Levy-driven OUP in the LM-input case.

**Power clock and power motions**. The clock ratio $C\left(t_{1}\right)/C\left(t_{2}\right)$ is a function of the temporal ratio $t_{2}/t_{1}$ alone—rather than a function of the time points $t_{1}$ and $t_{2}$—if and only if the clock is a power function: $C\left(t\right)=t^{\gamma }$, where γ is a positive exponent (as the clock is monotone increasing). In turn, the power clock produces extensions of power motions: an extension of power BM [134,135,136,137] in the BM-input case; and an extension of power LM [83,84,85] in the LM-input case. In both these extensions the underlying Hurst exponent H (of power BM and of power LM) is extended from the positive values (H>0) to the zero value (H=0).

## 6. Steady-State Insights

Section 5 introduced and analyzed the SSMs. This section carries on with further insights regarding the SSMs: the prediction of their increments (Section 6.1); their asymptotic behaviors (Section 6.2); and their ranges of dependence (Section 6.3).

### 6.1. Prediction

Denote by $\Delta =t_{2}-t_{1}$ the temporal lag between the time points $t_{1}$ and $t_{2}$ (where $t_{0}<t_{1}<t_{2}$). Keeping the time point $t_{1}$ fixed, note that (as the clock C(t) is monotone increasing): the larger the lag Δ—the smaller the clock-ratio $r=C\left(t_{1}\right)/C\left(t_{1}+\Delta \right)$. Thus, the different shapes of the functions $\phi \left(r\right)$ and $\phi_{\lambda }\left(r\right)$—which are specified in Table 2 and Table 3 above—imply the following behaviors of the corresponding quantities.

▶ **Increasing shape**: the larger the lag Δ—the smaller the quantity.
▶ **Decreasing shape**: the larger the lag Δ—the larger the quantity.
▶ **Flat shape**: the quantity is invariant with respect to the lag Δ.

For quantities #1 to #5, intuition suggest that the shape is increasing. Indeed, as the lag Δ grows larger, one would expect that: the correlations grow smaller; and the survival probability grows smaller. For these quantities intuition is correct.

Quantities #6 to #8 quantify the SSMs’ memory: how does the knowledge of the ‘present position’ $X\left(t_{1}\right)$ affect the prediction of the ‘future increment’ $X\left(t_{2}\right)-X\left(t_{1}\right)$. As with weather forecasts and with stock-market forecasts, intuition suggest that: the larger the lag Δ—the more ‘noisy’ the prediction. So, as the lag Δ grows larger: the SNR should grow smaller; and the NNR and the VVR should grow larger. Memory-wise intuition turns out to be tricky—as shall now be elucidated.

When the SLS exponent is in the range 1<λ≤2 then intuition is correct. However, when the SLS exponent is in the range 0<λ≤1 then intuition is wrong. Indeed, when the SLS exponent is in the range 0<λ<1 then the following counter-intuitive behavior is displayed: the larger the lag Δ—the better the prediction. And when the SLS exponent is one, λ=1, then the following odd behavior is displayed: the prediction is invariant with respect to the lag Δ.

The ‘prediction invariance’ holds only when λ=1, and this particular value of the SLS exponent marks a phase transition between the counter-intuitive prediction behavior (0<λ<1) and the intuitive prediction behavior (1<λ≤2). The SLS exponent λ=1 also characterizes the case in which the SLS input is the Cauchy process [90,91,92].

And there is more to the counter-intuitive and odd behaviors of the prediction. Equation (2) implies that the smaller the SLS exponent λ—the ‘heavier’ the tails of the SLS distribution, and hence the ‘wilder’ the distribution’s fluctuations. Consequently, intuition suggest that: the smaller the SLS exponent λ—the more ‘noisy’ the prediction. Yet again, intuition is wrong.

Keeping the SLS exponent fixed (at any value in the range 0<λ≤2), the shapes of quantities #6 to #8—as functions $\phi_{\lambda }\left(r\right)$ of the clock-ratio *r*—are specified in Table 3 above. Keeping the clock-ratio *r* fixed (at any value in the range 0<r<1), the shapes of quantities #6 to #8—now as functions $\phi_{\lambda }\left(r\right)$ of the SLS exponent λ—are as follows (where $\phi_{0}\left(r\right)=\lim_{\lambda \to 0}\phi_{\lambda }\left(r\right)$).

▶ **SNR**: monotone decreasing from $\phi_{0}\left(r\right)=\infty$ to $\phi_{2}\left(r\right)=\sqrt{\frac{1-\sqrt{r}}{1+\sqrt{r}}}$.
▶ **NNR**: monotone increasing from $\phi_{0}\left(r\right)=0$ to $\phi_{2}\left(r\right)=\sqrt{\frac{1+\sqrt{r}}{2}}$.
▶ **VVR**: monotone increasing from $\phi_{0}\left(r\right)=0$ to $\phi_{2}\left(r\right)=\frac{1+\sqrt{r}}{2}$.

**Figure 4:**
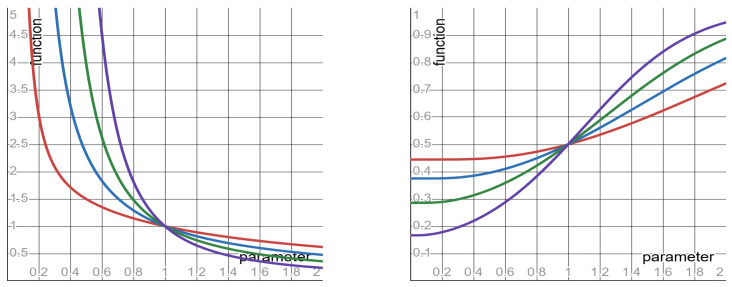
The functions $\phi_{\lambda }\left(r\right)$ specified in row #6 (**left panel**) and in row #8 (**right panel**) of Table 3, depicted with respect to their parameter λ. In both panels: red curve *r* = 0.2; blue curve *r* = 0.4; green curve *r* = 0.6; purple curve *r* = 0.8.

These monotone shapes (with respect to the SLS exponent λ) imply the following counter-intuitive behavior: the smaller the SLS exponent λ—the better the prediction. Namely, increasing the fluctuations of the SLS distribution (by decreasing the SLS exponent λ) results in a surprising outcome: the prediction becomes less noisy—rather than more noisy, as intuition suggests. The monotone shapes of the SNR and of the VVR are depicted in Figure 4.

### 6.2. Asymptotic Behaviors

As in Section 6.1, denote by $\Delta =t_{2}-t_{1}$ the temporal lag between the time points $t_{1}$ and $t_{2}$ (where $t_{0}<t_{1}<t_{2}$). Three asymptotic behaviors of the clock-ratio $r=C\left(t_{1}\right)/C\left(t_{1}+\Delta \right)$ shall now be addressed.

The first asymptotic behavior is with regard to the short-lag limit Δ→0. In this case the time point $t_{1}$ is kept fixed, and the resulting clock-ratio limit is one: $\lim_{\Delta \to 0}r=1$.

The second asymptotic behavior is with regard to the long-lag limit Δ→∞. In this case the time point $t_{1}$ is also kept fixed, and the resulting clock-ratio limit is zero: $\lim_{\Delta \to \infty }r=0$ (as the clock C(t) is monotone increasing to infinity).

The third asymptotic behavior is with regard to the large-time limit $t_{1}\to \infty$. In this case the temporal lag Δ is kept fixed, and the resulting clock-ratio limit is: either zero $\lim_{t_{1}\to \infty }r=0$ (as in the long-lag limit); or one $\lim_{t_{1}\to \infty }r=1$ (as in the short-lag limit); or intermediate, i.e., larger than zero and smaller than one. The intermediate scenario is described as follows.

In the intermediate scenario the clock-ratio limit admits a universal exponential form: $\lim_{t_{1}\to \infty }r=\exp\left(-\gamma \Delta \right)$, where γ is a positive exponent. This universal clock-ratio limit is the clock-ratio of the exponential clock: $C\left(t\right)=\exp\left(\gamma t\right)$. As noted in Section 5.3, the exponential clock produces the OUPs: the ‘regular’ OUP in the BM-input case; and the Levy-driven OUP in the LM-input case.

Introduce the function $C_{*}\left(u\right)=C\left[\ln\left(u\right)\right]$, where $u\geq \exp(t_{0})$. For the third asymptotic behavior, the clock-ratio limit is as follows [89]: zero, $\lim_{t_{1}\to \infty }r=0$, if and only if $C_{*}\left(u\right)$ is rapidly varying; one, $\lim_{t_{1}\to \infty }r=1$, if and only if $C_{*}\left(u\right)$ is slowly varying; and intermediate, $\lim_{t_{1}\to \infty }r=\exp\left(-\gamma \Delta \right)$, if and only if $C_{*}\left(u\right)$ is regularly varying—in which case γ is a positive ‘regular-variation exponent’.

So, the three asymptotic behaviors can be summarized as follows.

▶ The long-lag limit (Δ→∞), as well as the rapidly-varying scenario of the large-time limit ($t_{1}\to \infty$), yield the zero clock-ratio limit: r→0.
▶ The short-lag limit (Δ→0), as well as the slowly-varying scenario of the large-time limit ($t_{1}\to \infty$), yield the unit clock-ratio limit: r→1.
▶ The regularly-varying scenario of the large-time limit ($t_{1}\to \infty$) yields the OUP clock-ratio: $r=\exp\left(-\gamma \Delta \right)$, where γ is a positive exponent.

Per each of the eight quantities, Table 2 and Table 3 specify the limit values that correspond to the clock-ratio limits r→0 and r→1. Table 4 presents examples of clocks $C\left(t\right)$ that, in the large-time limit ($t_{1}\to \infty$), produce: the rapidly-varying scenario (r→0); and the slowly-varying scenario (r→1).

### 6.3. Range of Dependence

Consider a stationary process whose positions have finite variances. With no loss of generality, the process can be considered to be ‘standardized’, i.e.: its positions have zero means and unit variances. In turn, the process’ ‘second order statistics’ are coded by the process’ auto-correlation function $\rho \left(\Delta \right)$ (Δ≥0). Namely, $\rho \left(t_{2}-t_{1}\right)$ is the correlation of the process’ positions at the time points $t_{1}$ and $t_{2}$ (where $t_{1}\leq t_{2}$).

The process’ ‘range-of-dependence’ is determined by the integrability of its auto-correlation function at infinity [80,81,82]. Specifically, when the auto-correlation function is integrable at infinity then the process is said to be short-range dependent (SRD). And when the auto-correlation function is not integrable at infinity then the process is said to be long-range dependent (LRD). The integrability at infinity can be formulated as follows: fix the time point $t_{1}$, and check the integrability of $\rho \left(t_{2}-t_{1}\right)$—as a function of the temporal variable $t_{2}$—in the limit $t_{2}\to \infty$.

Now, switch from a general stationary process to the SSMs of Section 5. As described in Section 5.1 with regard to the BM-input case, the switch induces the following replacement: the stationary correlation $\rho \left(t_{2}-t_{1}\right)$ is replaced by the steady-state correlation $\sqrt{C\left(t_{1}\right)/C\left(t_{2}\right)}$. In turn, the notions of SRD and LRD follow naturally to the SSMs (generated by BM input): fix the time point $t_{1}$, and check the integrability of $\sqrt{C\left(t_{1}\right)/C\left(t_{2}\right)}$—as a function of the temporal variable $t_{2}$—in the limit $t_{2}\to \infty$.

In the LM-input case the variances of the output’s positions are either not defined or infinite. Thus, the notions of SRD and LRD cannot be carried on ‘as is’ from the BM-input case to the LM-input case. Nonetheless, alternative notions of SRD and LRD can be devised for the LM-input case. These alternative notions are based on the survival probability of the SSMs (generated by LM input): $P\left(t_{1};t_{2}\right)=C\left(t_{1}\right)/C\left(t_{2}\right)$.

The survival probability $P\left(t_{1};t_{2}\right)$—as a function of the variable $t_{2}$—is a tail distribution function over the range $t_{2}\geq t_{1}$. Namely, for a fixed time point $t_{1}$ (where $t_{1}>t_{0}$): $P\left(t_{1};t_{2}\right)$ is monotone decreasing from the value $\lim_{t_{2}\to t_{1}}P\left(t_{1};t_{2}\right)=1$ to the value $\lim_{t_{2}\to \infty }P\left(t_{1};t_{2}\right)=0$. In turn, the mean of the corresponding statistical distribution is: finite when the tail distribution function is integrable; and infinite when the tail distribution function is not integrable.

As the survival probability is the clock ratio, the integrability of the tail distribution function $P\left(t_{1};t_{2}\right)$ is equivalent to: the integrability of the function $1/C\left(t_{2}\right)$ over the range $t_{2}\geq t_{1}$ (where $t_{1}>t_{0}$). Thus, the notions of SRD and LRD—for SSMs that are generated by LM input—are determined by the integrability of the reciprocal of the clock function at infinity.

▶ If $1/C\left(t\right)$ is integrable at infinity (t→∞) then the SSM is SRD.
▶ If $1/C\left(t\right)$ is not integrable at infinity (t→∞) then the SSM is LRD.

Table 4 presents examples of clocks $C\left(t\right)$ that—according to the integrability criteria for the LM-input case—produce SSMs that are either SRD or LRD.

## 7. Steady-State Dynamics

Section 5 and Section 6 investigated various statistics of the SSMs. This section investigates the dynamics of the SSMs (Section 7.1), draws insights from the dynamics (Section 7.2 and Section 7.3), and then demonstrates the insights via examples (Section 7.4).

### 7.1. Langevin Dynamics

Due to its independent-increments property, the SLS process is Markov [40,41,42]. Due to its structure, the spatio-temporal transformation of Equation (3) maps Markov inputs to Markov outputs. So, as the input is set to be the SLS process—the output is Markov.

The spatio-temporal transformation of Equation (3) maps the input’s positions to the output’s positions. The corresponding map of the input’s velocities to the output’s velocities is [84]:

(19) $$\dot{X}\left(t\right)=\frac{\dot{A}\left(t\right)}{A\left(t\right)}X\left(t\right)+{\left[A{\left(t\right)}^{\lambda }\dot{C}\left(t\right)\right]}^{1/\lambda }\dot{L}\left(t\right)\;.$$

The input’s velocity process is Gaussian ‘white noise’ in the BM-input case (λ=2), and is Levy ‘white noise’ in the LM-input case (0<λ<2). [When the output’s range ($t\geq t_{0}$) stretches beyond the input’s range (τ≥0) then the white noise should be stretched accordingly.]

Equation (19) manifests *Langevin dynamics* that are ‘driven’ by the input process. More specifically, in the BM-input case Equation (19) is a ‘regular’ Langevin stochastic differential equation (SDE) [47,48,49]. And in the LM-input case Equation (19) is a ‘Levy-driven’ Langevin SDE—an object that attracted major interest [154,155,156,157,158,159,160,161,162,163,164,165], and that continues to do so [166,167,168,169,170,171,172,173,174].

The term $\dot{A}\left(t\right)/A\left(t\right)$ appearing on the right-hand side of Equation (19) is the *damping coefficient* of the Langevin dynamics. In the BM-input case (λ=2) the term $A{\left(t\right)}^{\lambda }\dot{C}\left(t\right)$ appearing on the right-hand side of Equation (19) is the *diffusion coefficient* of the Langevin dynamics. In general—and in analogy with the extended variance of Equation (4)—the term $A{\left(t\right)}^{\lambda }\dot{C}\left(t\right)$ can be addressed as an ‘extended diffusion coefficient’.

Consider the output to be in steady state, and—as in Section 5.2—set $A\left(t\right)=C{\left(t\right)}^{-1/\lambda }$. In turn: the damping coefficient is $-\frac{1}{\lambda }\dot{C}\left(t\right)/C\left(t\right)$; and the extended diffusion coefficient is $\dot{C}\left(t\right)/C\left(t\right)$. So, the Langevin dynamics of the SSMs are governed by the SLS exponent λ, and by the following infinite-dimensional parameter: the clock’s logarithmic derivative $\dot{C}\left(t\right)/C\left(t\right)$.

As the clock $C\left(t\right)$ is positive and monotone increasing (over the temporal range $t>t_{0}$), the damping coefficient is negative: $-\frac{1}{\lambda }\dot{C}\left(t\right)/C\left(t\right)<0$. In turn, the Langevin dynamics of the SSMs are ‘pushing’ towards the value zero—which is the median of the SSMs’ positions. So, the Langevin dynamics of the SSMs are ‘center reverting’ [175,176,177].

The ratio of the damping coefficient $-\frac{1}{\lambda }\dot{C}\left(t\right)/C\left(t\right)$ to the extended diffusion coefficient $\dot{C}\left(t\right)/C\left(t\right)$ is −1/λ. The damping-diffusion ratio −1/λ is the negative Hurst exponent of the SLS input [83], and it almost characterizes the SSMs. Indeed, a calculation shows that the damping-diffusion ratio −1/λ implies that: $A{\left(t\right)}^{-\lambda }=C\left(t\right)+k$, where *k* is an integration constant. In turn, when the integration constant is zero (k=0) then the amplitude-clock relation $A\left(t\right)=C{\left(t\right)}^{-1/\lambda }$ is attained.

The damping coefficient $-\frac{1}{\lambda }\dot{C}\left(t\right)/C\left(t\right)$ and the extended diffusion coefficient $\dot{C}\left(t\right)/C\left(t\right)$ are both constant—rather than time dependent—if and only if the clock is an exponential function: $C\left(t\right)=\exp\left(\gamma t\right)$, where γ is a positive exponent (as the clock is monotone increasing). As noted in Section 5.3, the exponential clock produces the OUPs: the ‘regular’ OUP in the BM-input case; and the Levy-driven OUP in the LM-input case.

### 7.2. Logarithmic-Derivative Perspective

As noted in Section 5.2 with regard to the SSMs: the transformation’s (infinite-dimensional) parameter is the clock $C\left(t\right)$. As described in Section 7.1 with regard to the Langevin dynamics of the SSMs: the dynamics’ (infinite-dimensional) parameter is the clock’s logarithmic derivative: $H\left(t\right)=\dot{C}\left(t\right)/C\left(t\right)$ (the reason for using the letter *H* to denote the logarithmic derivative shall become clear in Section 7.3 below).

With no loss of generality, the clock’s initial value $C\left(t_{0}\right)$ can be assumed to be positive, and the clock’s logarithmic derivative can be assumed to be integrable at the temporal origin $t_{0}$. [Indeed, if either of these assumptions does not hold then simply set a new temporal origin that is larger than $t_{0}$.] Consequently, in terms of the logarithmic derivative $H\left(t\right)$ the clock admits the formulation

(20) $$C\left(t\right)=C\left(t_{0}\right)\exp\left[\int_{t_{0}}^{t}H\left(u\right)du\right]\;.$$

Now, assume that the logarithmic derivative has a limit value $H\left(\infty \right)=\lim_{t\to \infty }H\left(t\right)$. As the clock $C\left(t\right)$ is monotone increasing, its logarithmic derivative $H\left(t\right)$ is positive, and hence: the limit value $H\left(\infty \right)$ is either zero, or positive, or infinite.

Equation (20) implies that the clock ratio admits the formulation $C\left(t_{1}\right)/C\left(t_{2}\right)=\exp\left[-\int_{t_{1}}^{t_{2}}H\left(u\right)du\right]$. With this formulation at hand, an asymptotic calculation implies that: the large-time limit $t_{1}\to \infty$ of the clock-ratio $r=C\left(t_{1}\right)/C\left(t_{1}+\Delta \right)$ (where the temporal lag $\Delta =t_{2}-t_{1}$ is kept fixed) is $\lim_{t_{1}\to \infty }r=\exp\left[-H\left(\infty \right)\Delta \right]$. In turn, the following characterization of the three scenarios of the large-time limit—which were stated in Section 6.2—is attained.

▶ The slowly-varying scenario holds if and only if $H\left(\infty \right)=0$.
▶ The regularly-varying scenario holds if and only if $0<H\left(\infty \right)<\infty$.
▶ The rapidly-varying scenario holds if and only if $H\left(\infty \right)=\infty$.

Introduce the function $H_{1}\left(t\right)=tH\left(t\right)$, and assume that it has a limit value $H_{1}\left(\infty \right)=\lim_{t\to \infty }H_{1}\left(t\right)$. As the limit value $H\left(\infty \right)$, also the limit value $H_{1}\left(\infty \right)$ is: either zero, or positive, or infinite. The limit value $H_{1}\left(\infty \right)$ turns out to determine the integrability of the clock’s reciprocal $1/C\left(t\right)$ at infinity [46]: if $H_{1}\left(\infty \right)<1$ then $1/C\left(t\right)$ is not integrable; and if $H_{1}\left(\infty \right)>1$ then $1/C\left(t\right)$ is integrable. In turn, the integrability criteria of Section 6.3 yield the following implications for SSMs that are generated by LM input.

▶ If $H_{1}\left(\infty \right)<1$ then the SSM is LRD.
▶ If $H_{1}\left(\infty \right)>1$ then the SSM is SRD.

Evidently, if $H\left(\infty \right)$ is either positive or infinite then $H_{1}\left(\infty \right)=\infty$. Thus, the following pair of conclusions holds for SSMs that are generated by LM input. LRD conclusion: the SSM can be LRD only in the slowly-varying scenario. SRD conclusion: in the regularly-varying scenario and in the rapidly-varying scenario the SSM is always SRD.

### 7.3. Hazard-Rate Perspective

It follows from Equation (20) that $C\left(t_{0}\right)/C\left(t\right)$ is a tail distribution function over the temporal range $t>t_{0}$. Namely, there is a random variable *T* that takes values in the temporal range $t>t_{0}$, and whose statistical distribution is governed by the tail distribution function

(21) $$\mathrm{Pr}\left(T>t\right)=\exp\left[-\int_{t_{0}}^{t}H\left(u\right)du\right]\;.$$

Equation (21) implies that the function $H\left(t\right)$ ($t>t_{0}$) is the *hazard rate* of the random variable *T* [178,179,180]. Namely, $H\left(t\right)$ is the likelihood that the random variable *T* be realized right after the time point *t*—given the information that *T* was not realized up to the time point *t*.

So, in terms of the random variable *T* the clock’s logarithmic derivative $H\left(t\right)$ admits a probabilistic hazard-rate meaning (and hence the letter *H* was used to denote the clock’s logarithmic derivative). Also, in terms of the random variable *T* the clock-ratio admits the following conditional-probability meaning:

(22) $$\frac{C\left(t_{1}\right)}{C\left(t_{2}\right)}=\mathrm{Pr}\left(T>t_{2}|T>t_{1}\right)\;.$$

Namely, the clock ratio is the probability that *T* is larger than $t_{2}$, given the information that *T* is larger than $t_{1}$ (where $t_{0}<t_{1}<t_{2}$).

Moreover, the integrability of the clock’s reciprocal $1/C\left(t\right)$ at infinity is equivalent to the convergence of the mean $E[T_{+}]$, where $T_{+}=\max\left\{0,T\right\}$ is the positive part of the random variable *T*. In turn, the following holds for SSMs that are generated by LM input: the SSM is SRD if and only if $E[T_{+}]<\infty$; and the SSM is LRD if and only if $E[T_{+}]=\infty$.

### 7.4. Examples

The logarithmic-derivative perspective and the hazard-rate perspective that were presented in Section 7.2 and Section 7.3 shall now be demonstrated via three examples: Pareto [181]; Weibull [182]; and Gumbel [183]. These examples correspond, respectively, to the following clock examples of Table 4: #1, #3, and #5. As in Table 4, in all three examples γ is a positive exponent.

Pareto distributions [184,185,186] and the Weibull distribution [187,188,189] are widely applied in science and engineering. The Weibull distribution and the Gumbel distributions are (two of the three) universal extreme-value distributions [190,191,192].

**Pareto example**. In this example $t_{0}$ is a positive number, and the tail distribution function is Pareto “type I”: $\mathrm{Pr}\left(T>t\right)={\left(t_{0}/t\right)}^{\gamma }$. In turn, the hazard rate is the harmonic function $H\left(t\right)=\gamma /t$, and hence: $H\left(\infty \right)=0$ and $H_{1}\left(\infty \right)=\gamma$. In this example the conditional probability $\mathrm{Pr}(T>t_{2}$|$T>t_{1})$ is also a Pareto “type I” tail distribution function.

**Weibull example.** In this example $t_{0}=0$, and the tail distribution function is Weibull: $\mathrm{Pr}\left(T>t\right)=\exp\left(-t^{\gamma }\right)$. In turn, the hazard rate is the power function $H\left(t\right)=\gamma t^{\gamma -1}$, and hence: $H\left(\infty \right)=0$ when γ<1; $H\left(\infty \right)=1$ when γ=1; $H\left(\infty \right)=\infty$ when γ>1; and $H_{1}\left(\infty \right)=\infty$. The hazard limits $H\left(\infty \right)=0$ and $H\left(\infty \right)=\infty$ characterize, respectively, the sub-exponential and the super-exponential ‘relaxation regimes’ of the Weibull distribution [193,194,195].

**Gumbel example**. In this example $t_{0}=-\infty$, and the tail distribution function is Gumbel: $\mathrm{Pr}\left(T>t\right)=\exp\left[-\gamma \exp\left(t\right)\right]$. In turn, the hazard rate is the exponential function $H\left(t\right)=\gamma \exp\left(t\right)$, and hence: $H\left(\infty \right)=\infty$ and $H_{1}\left(\infty \right)=\infty$. In this example the conditional probability $\mathrm{Pr}(T>t_{2}\;|\;T>t_{1})$ is the Gompertz tail distribution function [196,197,198].

## 8. Overview

This paper introduced and explored a novel and versatile class of real-valued random motions that are in statistical steady state. This section offers readers an ‘executive-summary’ overview of the paper.

The steady-state class is devised via a spatio-temporal transformation of a foundational real-valued random motion: the symmetric Levy-sable (SLS) process. The SLS process, its spatio-temporal transformation, and the resulting steady-state motions (SSMs) are as follows.

The SLS process is parameterized by an exponent λ that takes values in the range 0<λ≤2, and it comprises two random motions. Brownian motion (BM), which is characterized by the SLS exponent value λ=2. And Levy motion (LM), which is characterized by the SLS exponent range 0<λ<2.

BM and LM are principal stochastic models in science and engineering, and they display diametric behaviors. On the one hand BM is a continuous process, the variances of its positions are finite, and the probability tails of its positions are ‘light’. On the other hand LM is a pure-jump process, the variances of its positions are either not defined or infinite, and the probability tails of its positions are ‘heavy’. Thus, the fluctuations of BM are ‘mild’, and the fluctuations of LM are ‘wild’.

The spatio-temporal transformation is a mapping of real-valued random motions. The transformation acts by scaling, in both space and time, the positions of its input—thus generating the positions of its output. Here the input is the SLS process, and its positions are $L\left(\tau \right)$ (τ≥0). In turn, the output’s positions are

(23) $$X\left(t\right)=C{\left(t\right)}^{-1/\lambda }L\left[C\left(t\right)\right]$$

($t\geq t_{0}$), where $C\left(t\right)$ is the transformation’s clock.

The clock $C\left(t\right)$ is positive over the temporal range $t>t_{0}$, and it is monotone increasing to infinity: $\lim_{t\to \infty }C\left(t\right)=\infty$. The term $C{\left(t\right)}^{-1/\lambda }$ appearing on the right-hand side of Equation (23) is the transformation’s amplitude. The amplitude $C{\left(t\right)}^{-1/\lambda }$ determines the transformation’s spatial scaling, and the clock $C\left(t\right)$ determines the transformation’s temporal scaling.

The amplitude and the clock are coupled in a particular way: to produce SSMs. Namely, outputs whose positions—at all time points—share the same statistical distribution. And indeed, the properties of the SLS process imply that the output’s positions $X\left(t\right)$ are all equal, in law, to the random variable $L\left(1\right)$ (the input’s position at the time point τ=1).

Equation (23) maps the positions of the SLS to the positions of the SSMs. The mapping of the corresponding velocities is given by the following Langevin stochastic differential equation:

(24) $$\dot{X}\left(t\right)=-\frac{1}{\lambda }H\left(t\right)X\left(t\right)+{\left[H\left(t\right)\right]}^{1/\lambda }\dot{L}\left(t\right)\;,$$

where $H\left(t\right)=\dot{C}\left(t\right)/C\left(t\right)$ is the clock’s logarithmic derivative. The stochastic dynamics that are manifested by the Langevin Equation (24) are center reverting, i.e.: the dynamics are ‘pushing’ towards the origin 0 of the spatial axis. When the input is LM then the Langevin Equation (24) is ‘Levy-driven’.

With no loss of generality, the initial time point $t_{0}$ (of the temporal axis $t\geq t_{0}$) can be set so that the clock’s initial value is one: $C\left(t_{0}\right)=1$. Doing so, the clock’s reciprocal $1/C\left(t\right)$ and the clock’s logarithmic derivative $H\left(t\right)$ turn out to have probabilistic manifestations. These manifestations are in terms of a general random variable *T* that takes values over the temporal range $t>t_{0}$.

Indeed, the clock’s reciprocal and the clock’s logarithmic derivative are, respectively, the random-variable’s tail distribution function and hazard rate:

(25) $$\mathrm{Pr}\left(T>t\right)=\frac{1}{C\left(t\right)}=\exp\left[-\int_{t_{0}}^{t}H\left(u\right)du\right]\;.$$

Equation (25) couples together—vividly and transparently—the random variable *T*, the function $C\left(t\right)$, and the function $H\left(t\right)$.

So, the SSMs have two underlying parameters: the input’s and the transformation’s. The input’s parameter is the SLS exponent λ, it is one-dimensional, it governs the statistics of the SSMs’ positions, and hence it governs the SSMs’ fluctuations. The transformation’s parameter is infinite-dimensional, it governs the SSMs’ temporal structure, and hence it governs the SSMs’ inter-dependencies. The transformation’s parameter admits the following equivalent representations.

▶ A scaling representation via the function $C\left(t\right)$ of Equation (23).
▶ A Langevin representation via the function $H\left(t\right)$ of Equation (24).
▶ A probabilistic representation via the random variable *T* of Equation (25).

The SSMs were analyzed, comprehensively, via: eight different quantities; three different asymptotics; and a pair of integrability criteria. The eight quantities are specified in Table 2 and Table 3 above, and they comprise two sets. A set of five quantities that depend on the transformation’s parameter alone, and that quantify the SSMs’ inter-dependencies. And a set of three quantities that depend on both parameters, and that quantify the SSMs’ memory.

Focusing on temporal lags, the three asymptotics address (per each of the eight quantities): a short-lag limit; a long-lag limit; and a large-time limit. The integrability criteria depend on the transformation’s parameter alone, and they determine when the inter-dependencies of the SSMs are either short-ranged or long-ranged. The analysis provides a detailed ‘statistical picture’ of the SSMs.

Special cases of SSMs are Ornstein-Uhlenbeck processes (OUPs). Namely: the ‘regular’ OUP when the input is BM; and the ‘Levy-driven’ OUP when the input is LM. The OUPs are characterized by the following equivalent statements (in statements #3 to #5 γ is a positive Ornstein-Uhlenbeck parameter).

1. The SSM is a stationary process.
2. The Langevin Equation (24) is time homogeneous.
3. The clock is an exponential function, $C\left(t\right)=\exp\left(-\gamma t\right)$.
4. The hazard rate is a flat function, $H\left(t\right)=\gamma$.
5. The random variable *T* is exponentially distributed with mean E[T]=1/γ.

The regular OUP is the only random motion that is Gaussian *and* Markov *and* stationary. In the transition from the regular OUP to the Levy-driven OUP the Gaussian property is discarded, and the Markov and stationary properties are maintained. In the transition from the regular OUP to Gaussian-stationary processes the Markov property is discarded, and the Gaussian and stationary properties are maintained.

In the transition from the regular OUP to the SSMs the Markov property is maintained, the stationary property is relaxed, and the Gaussian property: is maintained in the BM-input case; and is discarded in the LM-input case. A comparison of the different stochastic models, and of their key features, is presented in Table 5.

In bottom line, this paper established a versatile stochastic model for real-valued random motions. The model has a one-dimensional parameter and an infinite-dimensional parameter, and it facilitates the combination of the following ‘regular’ and ‘anomalous’ features.

▶ **Regular-wise: the motions are in steady state, are Markov, and their dynamics are Langevin.**
▶ **Anomalous-wise, and tuned by the one-dimensional parameter: the motions can display wild fluctuations—a.k.a. ‘Noah effect’—and they have an adjustable memory structure.**
▶ **Anomalous-wise, and tuned by the infinite-dimensional parameter: the motions can display long-ranged temporal dependencies—a.k.a. ‘Joseph effect’—and they have an adjustable correlation structure.**

As noted in the introduction and along the manuscript: Levy-driven stochastic models in general—and, in particular, the Levy-driven OUP and the Levy-driven Langevin dynamics—attracted and attract major interest in science and engineering. With regard to the LM input: this paper established a Levy-driven stochastic model, with Levy-driven Langevin dynamics, and with the above features (see also the right column of Table 5).

Several potential directions for future research of the novel steady-state stochastic model are the following. For theoreticians: the study of the model’s first-passage times [199,200,201]; and the study of the model ‘under restart’ [202,203,204]. For statisticians: estimation of the model’s underlying parameters. And for experimentalists and practitioners: real-world applications of the model. Evidently, real-world applications and parameters estimation are intertwined. Future research in these intertwined directions can begin with special cases of the steady-state model, e.g., the ones presented in Table 4.

## Figures and Tables

**Table 1:** SLS distributions of the output’s increments $X\left(t_{2}\right)-X\left(t_{1}\right)$ (where $t_{0}<t_{1}<t_{2}$). The table’s rows correspond to: the unconditional distribution—in which case no information is provided; and the conditional distribution—in which case the information $X\left(t_{1}\right)$ is provided. The table’s columns correspond to: the median of the SLS distribution; and the extended variance of the SLS distribution—which is the $\lambda^{\mathrm{th}}$ power of the distribution’s scale.

	Median	Extended Variance
**Unconditional distribution**	0	$V\left(t_{1}\right){\left|\frac{A\left(t_{2}\right)}{A\left(t_{1}\right)}-1\right|}^{\lambda }+V\left(t_{2}\right)\left[1-\frac{C\left(t_{1}\right)}{C\left(t_{2}\right)}\right]$
**Conditional distribution**	$\left[\frac{A\left(t_{2}\right)}{A\left(t_{1}\right)}-1\right]X\left(t_{1}\right)$	$V\left(t_{2}\right)\left[1-\frac{C\left(t_{1}\right)}{C\left(t_{2}\right)}\right]$

**Table 2:** Quantities #1 to #5—of the SSMs—as functions $\phi \left(r\right)$ of the clock-ratio $r=C\left(t_{1}\right)/C\left(t_{2}\right)$ (where $t_{0}<t_{1}<t_{2}$). Per each quantity, the table specifies: the function $\phi \left(r\right)$ and its limit values, $\phi \left(0\right)=\lim_{r\to 0}\phi \left(r\right)$ and $\phi \left(1\right)=\lim_{r\to 1}\phi \left(r\right)$.

Quantity	Function $\phi \left(r\right)=\;$	Limit $\phi \left(0\right)=\;$	Limit $\phi \left(1\right)=\;$
(**1**) $F_{BM}\left(t_{1},t_{2}\right)$	$\sqrt{r}$	0	1
(**2**) $G_{BM}\left(t_{1},t_{2}\right)$	$-\sqrt{\frac{1-\sqrt{r}}{2}}$	$-\frac{1}{\sqrt{2}}$	0
(**3**) $P\left(t_{1},t_{2}\right)$	*r*	0	1
(**4**) $F_{LM}\left(t_{1},t_{2}\right)$	*r*	0	1
(**5**) $G_{LM}\left(t_{1},t_{2}\right)$	$-\sqrt{\frac{1-r}{2}}$	$-\frac{1}{\sqrt{2}}$	0

**Table 3:** The mother quantity $Q\left(t_{1};t_{2}\right)$ and quantities #6 to #8—of the SSMs—as functions $\phi_{\lambda }\left(r\right)$ of the clock-ratio $r=C\left(t_{1}\right)/C\left(t_{2}\right)$ (where $t_{0}<t_{1}<t_{2}$). Per each quantity, the table specifies: the function $\phi_{\lambda }\left(r\right)$ and its shape—which is determined by the value of the SLS exponent λ. The notation a↗b is shorthand for: monotone increasing from the level $a=\lim_{r\to 0}\phi_{\lambda }\left(r\right)$ to the level $b=\lim_{r\to 1}\phi_{\lambda }\left(r\right)$. The notation b↘a is shorthand for: monotone decreasing from the level $b=\lim_{r\to 0}\phi_{\lambda }\left(r\right)$ to the level $a=\lim_{r\to 1}\phi_{\lambda }\left(r\right)$.

Quantity	Function $\phi_{\lambda }\left(r\right)=\;$	λ<1	λ=1	λ>1
$Q\left(t_{1};t_{2}\right)$	$\frac{{\left(1-r^{1/\lambda }\right)}^{\lambda }}{1-r}$	1↗∞	$\phi_{1}\left(r\right)=1$	1↘0
(**6**) $SNR\left(t_{1};t_{2}\right)$	$\frac{1-r^{1/\lambda }}{{\left(1-r\right)}^{1/\lambda }}$	1↗∞	$\phi_{1}\left(r\right)=1$	1↘0
(**7**) $NNR\left(t_{1};t_{2}\right)$	${\left[\frac{1-r}{1-r+{\left(1-r^{1/\lambda }\right)}^{\lambda }}\right]}^{1/\lambda }$	$\frac{1}{2^{1/\lambda }}↘0$	$\phi_{1}\left(r\right)=\frac{1}{2^{1/\lambda }}$	$\frac{1}{2^{1/\lambda }}↗1$
(**8**) $VVR\left(t_{1};t_{2}\right)$	$\frac{1-r}{1-r+{\left(1-r^{1/\lambda }\right)}^{\lambda }}$	$\frac{1}{2}↘0$	$\phi_{1}\left(r\right)=\frac{1}{2}$	$\frac{1}{2}↗1$

**Table 4:** Examples of clocks $C\left(t\right)$ ($t\geq t_{0}$). In all examples γ is a positive exponent. With regard to the large-time limit ($t_{1}\to \infty$) of Section 6.2, and per each clock: the ‘r→0’ column and the ‘r→1’ column specify, respectively, if and when the rapidly-varying scenario and the slowly-varying scenario are attained. With regard to the integrability criteria of Section 6.3 (for the LM-input case), and per each clock: the SRD column and the LRD column specify, respectively, if and when the SSM is either SRD or LRD.

Clock	Origin	r→0	r→1	SRD	LRD
(**1**) $C\left(t\right)=t^{\gamma }$	$t_{0}=0$	no	yes	γ>1	γ≤1
(**2**) $C\left(t\right)=\ln{\left(t\right)}^{\gamma }$	$t_{0}=1$	no	yes	γ>1	γ≤1
(**3**) $C\left(t\right)=\exp\left(t^{\gamma }\right)$	$t_{0}=0$	γ>1	γ<1	yes	no
(**4**) $C\left(t\right)=\exp\left[\ln{\left(t\right)}^{\gamma }\right]$	$t_{0}=1$	γ>1	γ≤1	γ>1	γ≤1
(**5**) $C\left(t\right)=\exp\left[\gamma \exp\left(t\right)\right]$	$t_{0}=-\infty$	yes	no	yes	no

**Table 5:** Comparison of stochastic models. The table’s columns correspond to the following models: ‘regular’ OUP; ‘Levy-driven’ OUP; Gaussian-stationary processes; Brown SSM (which is generated by BM input); and Levy SSM (which is generated by LM input). Per each model, the table’s rows address the following questions. (**1**) What are the model’s parameters? (**2**) Is the model Gaussian? (**3**) Is the model Markov (with Langevin dynamics)? (**4**) Is the model stationary? (**5**) Can the model display the Noah effect? (**6**) Can the model display the Joseph effect? (**7**) Does the model have an adjustable correlation structure? (**8**) Does the model have an adjustable memory structure? Regarding the OUPs: γ is a positive OUP parameter. Regarding the Gaussian-stationary processes (see [76] for the results per this model): $\rho \left(\Delta \right)$ (Δ≥0) is the auto-correlation function.

	OUP	LOUP	GS	BSSM	LSSM
**(1) Parameters**	γ	λ,γ	$\rho \left(\Delta \right)$	*T*	λ,T
**(2) Gaussian**	yes	no	yes	yes	no
**(3) Markov**	yes	yes	no	yes	yes
**(4) Stationary**	yes	yes	yes	semi	semi
**(5) Noah**	no	yes	no	no	yes
**(6) Joseph**	no	no	yes	yes	yes
**(7) Correlation**	no	no	yes	yes	yes
**(8) Memory**	no	yes	no	no	yes

## Data Availability

No new data were created or analyzed in this study.

## Appendix A

Consider a pair of real-valued random variables, $Z_{1}$ and $Z_{2}$, that satisfy the following conditions: same variances, $\mathrm{Var}\left[Z_{1}\right]=\mathrm{Var}\left[Z_{2}\right]=v$, where v>0; and correlation *c*, where −1≤c<1. Then, the correlation of the random variable $Z_{1}$ and the random-variables difference $Z_{2}-Z_{1}$ is:

(A1) $$\left.\begin{array}{c} \frac{\mathrm{Cov}\left[Z_{1},Z_{2}-Z_{1}\right]}{\sqrt{\mathrm{Var}[Z_{1}]}\sqrt{\mathrm{Var}[Z_{2}-Z_{1}]}}\\ \;\\ =\frac{\mathrm{Cov}\left[Z_{1},Z_{2}\right]-\mathrm{Var}\left[Z_{1}\right]}{\sqrt{\mathrm{Var}[Z_{1}]}\sqrt{\mathrm{Var}\left[Z_{1}\right]+\mathrm{Var}\left[Z_{2}\right]-2\mathrm{Cov}\left[Z_{1},Z_{2}\right]}}\\ \;\\ =\frac{cv-v}{\sqrt{v}\sqrt{2v-2cv}}=\frac{c-1}{\sqrt{2(1-c)}}\\ \;\\ =-\sqrt{\frac{1-c}{2}}\;.\; \end{array}\right.$$

Consider the SLS input to be BM (λ=2), and set $Z_{1}=X\left(t_{1}\right)$ and $Z_{2}=X\left(t_{2}\right)$ (where $t_{0}<t_{1}<t_{2}$). When the output is in steady state then the above conditions are met, and $c=F_{BM}\left(t_{1},t_{2}\right)$. Hence Equation (A1) implies that

(A2) $$G_{BM}\left(t_{1},t_{2}\right)=-\sqrt{\frac{1-F_{BM}\left(t_{1},t_{2}\right)}{2}}\;.\;$$

Consider the SLS input to be LM (0<λ<2), and set $Z_{1}=N_{l}\left(t_{1}\right)$ and $Z_{2}=N_{l}\left(t_{2}\right)$ (where $t_{0}<t_{1}<t_{2}$). When the output is in steady state then the above conditions are met, and $c=F_{LM}\left(t_{1},t_{2}\right)$. Hence Equation (A1) implies that

(A3) $$G_{LM}\left(t_{1},t_{2}\right)=-\sqrt{\frac{1-F_{LM}\left(t_{1},t_{2}\right)}{2}}\;.\;$$
